# Rebound Thrombocytosis after Induction Chemotherapy is a Strong Biomarker for Favorable Outcome in AML Patients

**DOI:** 10.1097/HS9.0000000000000180

**Published:** 2019-03-20

**Authors:** Bianca R. Schnell, Katja Seipel, Ulrike Bacher, Barbara Jeker, Beatrice U. Mueller, Yara Banz, Urban Novak, Thomas Pabst

**Affiliations:** 1 Department of Medical Oncology; 2 Department of Biomedical Research; 3 Department of Hematology and Central Hematology Laboratory, Inselspital, Bern University Hospital, University of Bern, Bern, Switzerland; 4 Institute of Pathology, University of Bern, Bern, Switzerland; 5 Center for Hemato-Oncology, University Cancer Center Inselspital, Bern University Hospital, University of Bern, Bern, Switzerland.

## Abstract

Whereas the molecular events underlying acute myeloid leukemia (AML) are increasingly identified, dynamics of hematologic recovery following induction chemotherapy remain mysterious. Platelet recovery may vary between incomplete and excess recovery among patients achieving remission. We analyzed platelet recovery after the first induction cycle in 291 consecutive AML patients. We defined excess platelet rebound (EPR) as platelet increase above 500 G/L. We observed EPR in 120 (41.2%) patients. EPR+ patients had lower platelets at diagnosis, higher marrow infiltration, more frequently *NPM1* mutations, and were associated with ELN favorable risk. Absence of EPR correlated with complex karyotypes, ELN intermediate-I and adverse risk, and therapy-related AML. Overall survival was better in EPR+ patients than EPR- (median 125 vs 41 months; p = 0.04), as was disease-free survival. By multivariate analysis, EPR+ was an independent parameter associated with favorable survival. Plasma thrombopoietin (TPO) levels at diagnosis indicated EPR+ (p < 0.0001), while *GATA-1*, *GATA-2,* and *MPL* mRNA expression did not differ between EPR+ and EPR- patients. Finally, transcription factors blocking early megakaryopoiesis were upregulated in EPR- patients, while *NFE2* involved in late megakaryocyte differentiation was increased in EPR+ patients. Our work identifies mechanisms involved in platelet recovery after induction chemotherapy.

## Introduction

Risk stratification in acute myeloid leukemia (AML) patients undergoing intensive chemotherapy is predominantly relying on disease characteristics obtained pre-treatment.^1–3^ Among them, current classification systems acknowledge disease-specific molecular and cytogenetic abnormalities as the major determinants.^4–6^ In contrast, clinical parameters at diagnosis of AML such as the degree of blast infiltration in the bone marrow and peripheral blood, presence and degree of cytopenias or leukocytosis, chemical parameters such as LDH, presence of extramedullary disease, or deregulated expression of cell surface markers hardly affect therapeutic decisions nowadays if at all.^2–7^ Whereas combined molecular and cytogenetic parameters allow risk stratification in the routine and in the clinical trial setting, they ultimately represent a wide range as far as the outcome of individual patients is concerned. Clearly, additional parameters are needed to improve outcome prediction in individual AML patients.

Data gathered after the start of AML treatment can potentially be equally useful as pre-treatment characteristics. For example, Keating et al suggested that the number of courses needed to achieve CR was inversely related to remission duration, and achievement of early remission (after the first induction cycle) is increasingly recognized as an important favorable prognostic determinant.^8^ Similarly, the depth of response to induction treatment assessed by quantitative determination of molecular and/or immunophenotypic leukemia-specific marker profiles is also of important prognostic relevance.^9–15^ In addition, next generation sequencing (NGS) has allowed for the identification of persisting molecular mutations in remission samples, with some of them predicting higher risk of relapse.^16^ Other parameters obtained during treatment with favorable impact on relapse risk may include the decrease of the stem cell mobilization potential, delayed hematologic recovery after consolidation treatment, and low levels of transfusion-dependent iron load.^17–19^

In this study, we assessed whether inter-individually differing kinetics of platelet recovery after induction chemotherapy may also confer relevant prognostic information. Thrombocytopenia is a common finding at diagnosis of AML, with surprisingly poor correlation to the degree of bone marrow infiltration.^20–21^ Consequently, it was suggested that thrombocytopenia at diagnosis of AML depends on deregulated cytokine expression in specific leukemia subtypes rather than on the degree of bone marrow infiltration per se.^20–21^ Similarly, recovery of platelets after the first induction cycle varies widely among individual patients even if morphologic remission is achieved. In particular, excess platelet rebound (EPR) with values exceeding 500 G/L can be observed in some patients. However, this clinical observation of EPR is poorly understood so far, and its prognostic relevance is unknown. In this study, we investigated whether EPR is associated with distinct subtypes of AML and whether it confers specific prognostic information.

## Patients and methods

### Patients and samples

In this single-center retrospective analysis, we investigated consecutive adult AML patients, who received intensive induction chemotherapy at the University Hospital of Berne, Switzerland between 01/2000 and 12/2016. We included untreated patients with de novo AML and secondary (therapy-associated or evolving from previous hematologic conditions) AML. We excluded patients receiving palliative treatment from this analysis, as well as patients, who were refractory to the first induction cycle and underwent a premature start of the second induction cycle, since platelet recovery could not be assessed in these patients. Informed consent from all patients was obtained according to the *Declaration of Helsinki*, and the study had approval by a decision of the local ethics committee of Bern, Switzerland (decision number #223/15). Molecular screening for *FLT3* (ITD and TKD), *NPM1*, and *TP53* mutations, and conventional cytogenetic analysis of at least 20 metaphases was available in all patients. We collected peripheral blood plasma, peripheral blood mononuclear cells (PBMCs) and bone marrow mononuclear cells (BMMCs) at the time of diagnosis before initiation of treatment.

### Treatment

Patients were treated in or according to the SAKK/HOVON-42, -43, -92, -102, -103, or -132 protocols. Briefly, patients received intravenously cytarabine 200 mg/m^2^ on days 1 to 7 and idarubicine 12 mg/m^2^ days 1 to 3 in cycle 1; and cytarabine 1000 mg/m^2^/q12 h days 1 to 6 and amsacrine 120 mg/m^2^ days 1 to 3 (until 12/2013; thereafter, daunorubicin 60 mg/m^2^ days 1, 3, 5) were given in cycle 2. For consolidation, allogeneic hematopoietic stem cell transplantation was offered to poor risk patients (with a sibling or an unrelated matched donor) and to intermediate risk patients (with a sibling donor). The remaining patients in CR1 preferentially received busulfan / cyclophosphamide high-dose chemotherapy with autologous transplantation, or (in case of failed stem cell collection) a third cycle of conventional chemotherapy with mitoxantrone and etoposide.^22^

### Definitions

The reference platelet count at our laboratory is 150 to 450 G/L. Patients were considered to have excess platelet rebound (EPR) in this study when the number of platelets exceeded 500 G/L after the first induction chemotherapy cycle, and intervals to EPR were counted starting from the first day of the first induction therapy. All patients had undergone a previous chemotherapy-related phase of thrombocytopenia before eventual subsequent EPR. EPR was considered as a parameter independent from the platelet level at diagnosis of the AML. We compared AML patients with EPR (EPR+) to AML patients without EPR (EPR−). Concurring severe infection as a possible cause for increased platelets counts at the respective time point was excluded in all patients, who finally were considered for the analysis.

Risk assessment followed the European Leukemia Net (ELN) classification as published in 2010.^3^ We used response criteria according to the International Working Group for diagnosis, standardization of response criteria, treatment outcomes, and reporting standards for therapeutic trials in AML.^23^ Bone marrow examinations were performed at days 18 and 28 of each induction cycle.

Progression free survival was calculated from the date of CR1 until disease progression, death or last follow-up, whichever occurred first. Non-relapsing alive patients were censored at the last date of follow up. Overall survival was calculated from the date of CR1 until death or last follow-up. Patients still alive or lost to follow-up were censored at the last date they were known to be alive.

### Statistical analysis

We calculated curves depicting progression-free survival and overall survival according to the Kaplan–Meier method. Survival analyses were performed using the log-rank method. Hazard ratios to evaluate the impact of baseline characteristics on clinical outcome were calculated using the log-rank method. All reported p values were from two-tailed Fisher's or unpaired *t* tests, and a value of p < 0.05 was considered to be statistically significant. Comparison of survival was performed using the log-rank method. The statistical analysis applied GraphPad Prism® Version 6.0 (GraphPad Software, La Jolla, CA).

### Gene expression analysis

RNA was extracted from AML cells and quantified using qPCR. The RNA extraction kit was supplied by Macherey-Nagel, Düren, Germany. Reverse transcription was done with MMLV-RT (Promega, Madison, WI). Real-time PCR was performed on the ABI7500 Real-Time PCR Instrument using FAST Start Universal probe master mix (Roche, Switzerland). The following gene specific probes were used (cat# 4331182, Thermo Fisher Scientific, Waltham, MA): Hs00180489_m1 for *MPL*; Hs00920556_m1 for *MYB*; Hs00232351_m1 for *NFE2*; Hs00958846_m1 for *NF1A*; Hs01085823_m1 for *GATA1*; Hs00231119_m1 for *GATA2*; Hs00358836_m1 for *KLF4*; and Hs02758991_g1 for *GAPDH*. *MPL, MYB, NFE2, NF1A, GATA1, GATA2* and *KLF4* Ct values were normalized with *GAPDH* Ct values (ddCt relative quantitation). Assays were performed in four physical replicates each. Statistical analysis used GraphPad Prism software for column analysis applying Mann-Whitney tests. Data in scatter plots include median values.

### Enzyme-linked immunosorbent assay (ELISA)

Thrombopoietin (*TPO*) levels were quantified in peripheral blood plasma at diagnosis using the human *TPO* ELISA kit (EHTHPO, Thermo Fisher Scientific). MPL protein levels were quantified in whole cell extracts of peripheral blood or bone marrow mononuclear cells obtained at first diagnosis using the human MPL ELISA kit (OKCD07412, Aviva Systems Biology, San Diego, CA). Whole cell extracts were prepared by lysis in RIPA buffer on ice for 60 minutes and centrifugation for 5 minutes. Protein extracts were diluted in TBS (1:100), and protein concentration was quantified by absorbance measurement A280 on NanoDrop 2000 spectrophotometer (Thermo Fisher Scientific). MPL protein concentration was normalized to total protein concentration. Plasma proteins and cellular protein extracts were assessed in three physical replicates each. Statistical analysis was performed on GraphPad Prism software using Mann-Whitney tests in column analysis. Data with scatter plots include median values.

## Results

We retrospectively studied a cohort of 291 consecutive patients with newly diagnosed AML, who received intensive standard induction chemotherapy at the University Hospital Berne, Switzerland, between 01/2000 and 12/2016. We excluded AML patients with palliative treatment and patients, who were refractory to the first induction cycle and underwent a premature start of the second induction cycle. Patients were analyzed according to their maximum platelet value observed during hematologic recovery after induction cycle 1. We found that 120 patients (41.2%) had maximum platelet values exceeding 500 G/L at least once and thus fulfilled the criteria for EPR (EPR+), while maximum platelet levels did not reach 500 G/L in 171 patients (58.8%; EPR−). The clinical characteristics at initial diagnosis of AML of EPR+ patients compared to EPR− patients are summarized in Table 1.

**Table 1:**
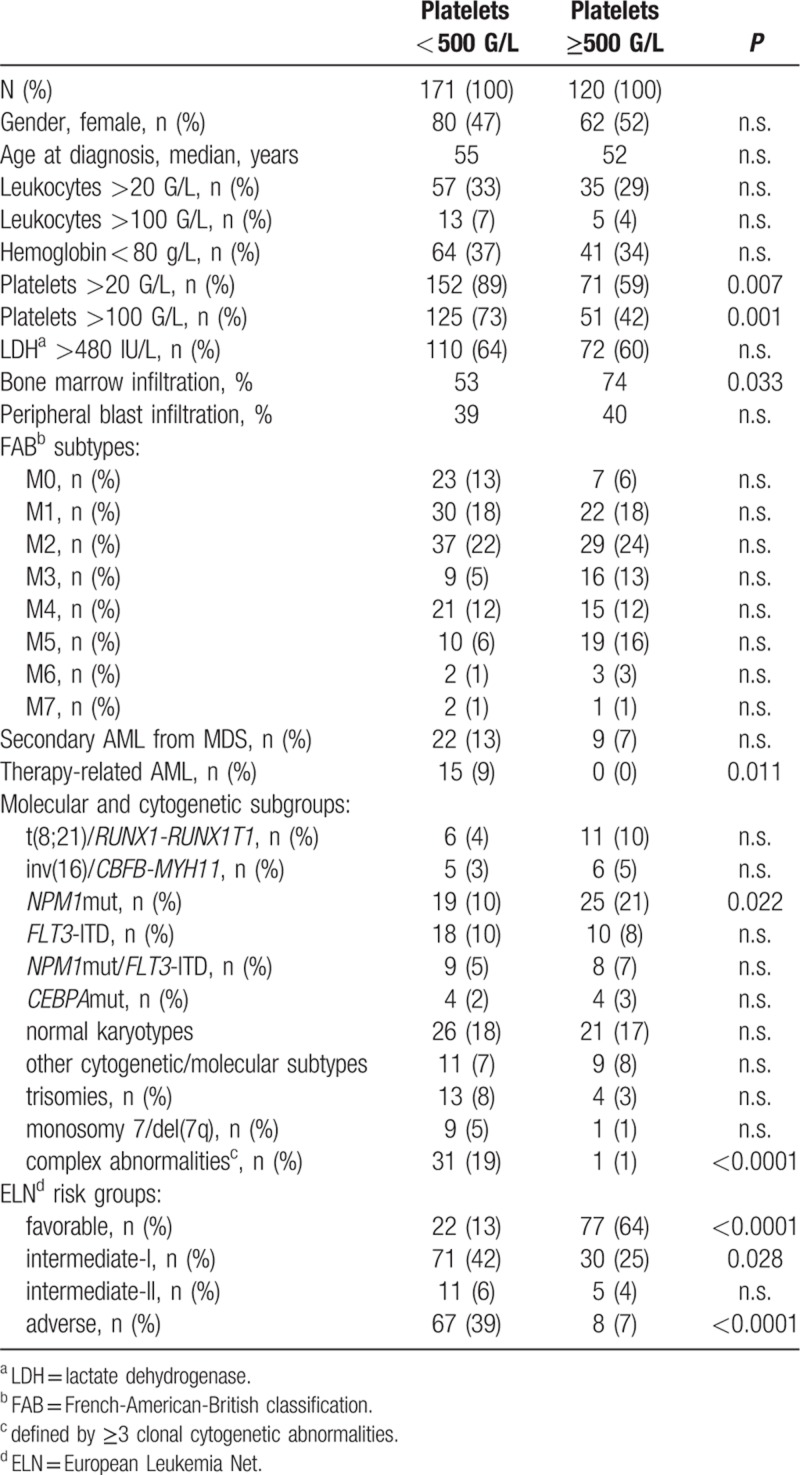
Patient characteristics at diagnosis of AML

### Dynamics of platelet recovery

Across all patients of our study cohort, platelet levels reached their maximum value after a median of 32 days after the start of the first induction cycle. Individual values are depicted in supplemental Figure 1, panel A, (Supplemental Digital Content). After the start of the second induction cycle, the median duration until maximum platelet levels was longer with 37 days (supplemental Fig. 1, panel B, Supplemental Digital Content). The maximum platelet levels after the first induction cycle ranged between 17 G/L and 1,416 G/L, with a median of 433 G/L (Supplemental Fig. 1; panel C, Supplemental Digital Content). After the second treatment cycle, the top platelet levels ranged between 19 G/L and 1020 G/L with a median of 248 G/L as shown in panel D in the supplemental Figure 1 (Supplemental Digital Content). Thus, the maximum platelet levels were higher after the first induction course as compared to the second induction course.

### Correlation studies with other peripheral blood values, laboratory parameters, and FAB subtypes

The degree of leukocytosis or anemia at initial presentation was not associated with EPR after induction cycle 1, whereas the degree of thrombocytopenia was inversely correlated with the occurrence of EPR+. EPR+ patients were more likely to have mild thrombocytopenia (<100 G/L) or strong thrombocytopenia (<20 G/L) at diagnosis of AML. Age, gender, LDH or FAB subtypes were not associated with developing EPR+. Remarkably, none of the 15 patients with therapy-related AML (p = 0.011) and only 9 of 31 patients (p = 0.059) with secondary AML evolving from previous MDS showed EPR after cycle 1.

### Correlation with inflammation parameters

We tried to correlate the occurrence of EPR+ with increased levels of C-reactive protein (CRP). Patients were separated according to a cut-off of 25 mg/L for CRP (reference level in our laboratory was < 5 mg/L). We selected the threshold of 25 mg/L for CRP, as this was the median CRP level observed in the total cohort at the time of hematologic recovery after the first induction cycle. In fact, platelet levels and the frequency of EPR+ were not significantly different in patients with CRP level ≥25 mg/L as compared to patients with CRP level < 25 mg/L (data not shown). These data do not support an association between EPR+ and an inflammatory state in our cohort.

### Association of maximum platelet levels with cytogenetic and molecular subgroups

Among the cytogenetic subgroups, patients with complex karyotype abnormalities were strongly associated with failing to have EPR, with only 1 EPR+ patient among 32 patients (p < 0.0001). Most likely, due to the small numbers, no differences between EPR+ and EPR− were observed for subgroups with other cytogenetic aberrations (Table 1). The median maximum platelet levels between cytogenetic subgroups ranged between 630 G/L in patients with t(8;21)/*RUNX1-RUNX1T1* and 140 G/L in patients with chromosome 7 alterations or with complex karyotype abnormalities (p = 0.0363 and p = 0.0018, respectively).

**Figure 1:**
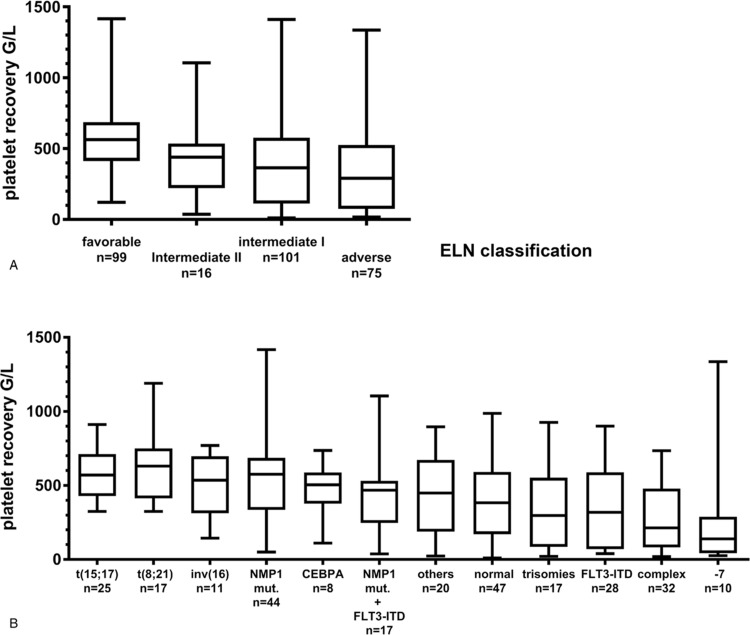
(A) Maximum platelet levels are depicted as observed during hematologic recovery after induction chemotherapy cycle 1 for the four ELN risk groups (following the 2010 publication). (B) The maximum platelet levels are shown as observed during hematologic recovery after induction chemotherapy cycle 1 for the various molecular and cytogenetic subgroups.

Moreover, among AML subgroups defined by molecular abnormalities, we identified patients with *NPM1* mutations (in the absence of a *FLT3* mutation) to be correlated with having EPR+ after induction cycle 1. We identified no significant differences for the other molecular markers, again, probably due to small patient numbers within these subgroups (Table 1). The median values and the distribution of individual values for each molecular subgroup are depicted in Figure 1. Across subgroups, significant differences were observed such as for patients with *NPM1* mutation (with *FLT3* wild-type), who had higher median platelet values (575 G/L) as compared to, for example, patients with chromosome 7 alterations or complex karyotypes (p = 0.0371 and p = 0.0002, respectively).

When applying the ELN risk classification, patients in the favorable risk category presented significantly more often with EPR+ after cycle 1 (p < 0.0001), whereas patients with intermediate-I (p = 0.028) or adverse risk (p < 0.0001) predominantly had no EPR. The median maximum platelet value after induction cycle 1 was 563 G/L in ELN favorable-risk patients as compared to 366 G/L in intermediate-I (p = 0.001) or to 292 G/L in adverse risk patients (p < 0.0001), whereas differences between intermediate-I and adverse-risk patients were not significant. Median values and distribution of individual platelet values for the ELN risk groups are depicted in Figure 1.

### Clinical outcomes in correlation to the maximum platelet levels

**Figure 2:**
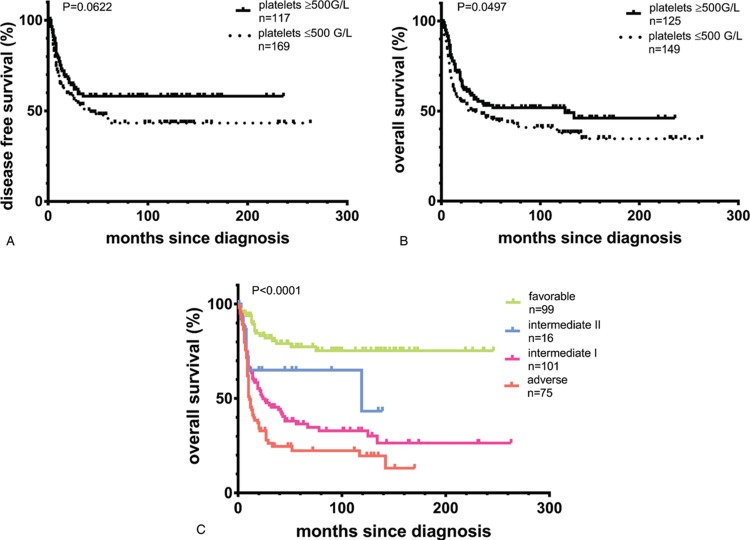
(A) Kaplan-Meier curves are presented for disease-free survival comparing AML patients with excess platelet rebound above 500 G/L after induction cycle 1 (EPR+) compared to EPR− patients. (B) Kaplan-Meier curves are depicted for overall survival comparing AML patients with EPR after induction cycle 1 (EPR+) compared to EPR− patients. (C) Kaplan-Meier curves are shown for overall survival comparing the four ELN risk groups of the study cohort.

The median disease-free survival was significantly lower with 38 months in the 171 patients without EPR as compared to not being reached in the 120 EPR+ patients (p = 0.0422; Fig. 2). Relapse in EPR+ and EPR− patients both occurred after a median of 13 months. Also, the median overall survival of EPR− patients was worse with 42 months compared to 125 months in EPR+ patients (p = 0.0497). Supplementary Figure 2 (Supplemental Digital Content) indicates that the survival rates showed no significant differences when EPR− and EPR+ patients were compared within the four ELN risk categories. This may also be related to the limited sample sizes of the different subgroups.

Furthermore, we excluded the possibility that survival differences are more pronounced when even more stringent criteria for the definition of EPR+ were applied: using a definition of EPR above vs below 700 G/L, overall survival did not differ significantly between the 2 groups (Supplementary Fig. 3, Supplemental Digital Content). Finally, in a multivariate analysis, EPR after induction cycle 1 was an independent prognostic factor associated with favorable overall survival together with age below vs above 60 years at diagnosis and favorable ELN risk group (vs intermediate-I/-II and adverse risk). In contrast, gender as well as peripheral leukocytes and platelet values at diagnosis of the AML had no independent prognostic impact by multivariate analysis (Table 2).

**Table 2:**
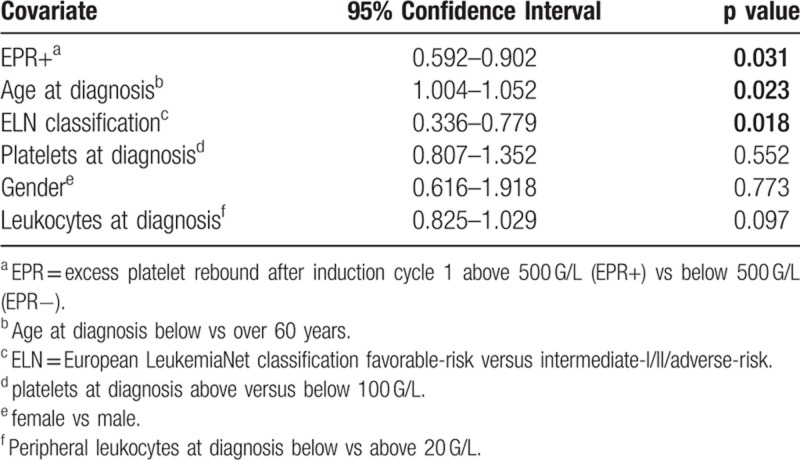
Multivariate Cox proportional hazard model for overall survival adjusting for risk factors

### Experimental studies investigating the pathophysiologic background of the level of platelet recovery

**Figure 3:**
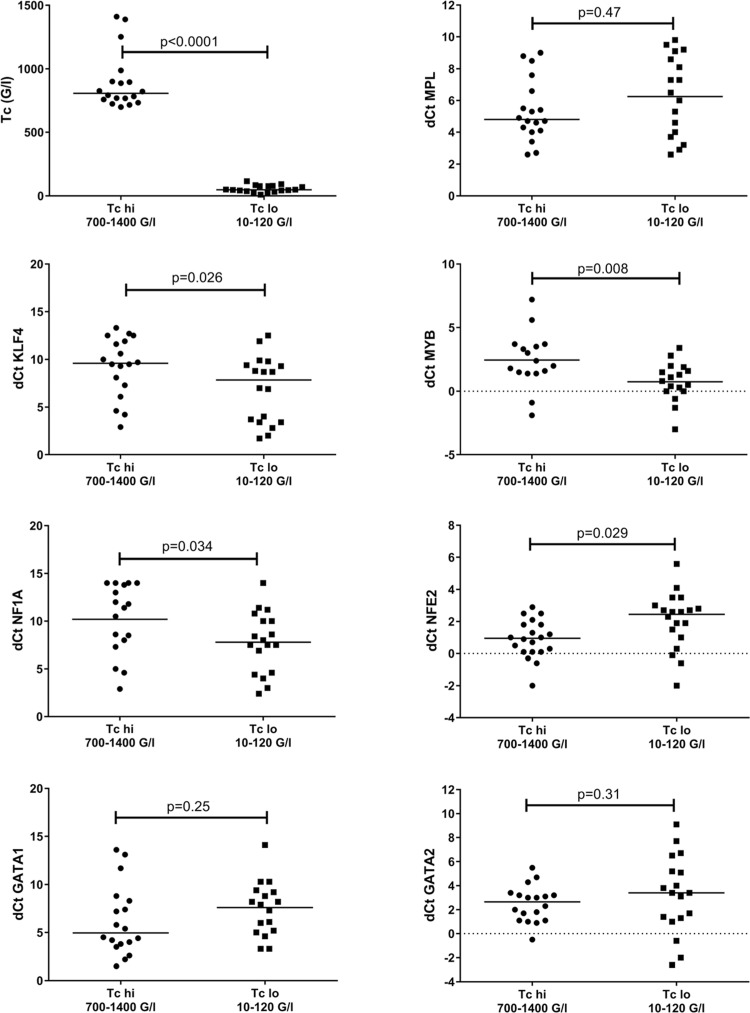
Samples at diagnosis of the 50 AML patients with maximum values (TOP50: 700–1,400 G/L) of excess platelet rebound (EPR) were compared to the 50 patients with the lowest maximum number of platelets (BOT50: 10–120 G/L) after induction chemotherapy cycle 1. (A) Thrombopoietin (TPO) was assessed in the plasma by ELISA, and MPL protein was determined using lysates from leukemic blasts. Peripheral blood from healthy volunteers is shown as a control. (B) mRNA expression using lysates from leukemic blasts at diagnosis in the two patient subgroups are presented as dCt values. The median dCt values are not significantly different for *MPL* gene expression, but they differ significantly for *KLF4*, *MYB*, *NF1A* and *NFE2*. There are no significant differences for *GATA1* and *GATA2* gene expression.

In order to elucidate the underlying mechanism for EPR, we compared data acquired in the 50 AML patients with the highest platelet values (range 700 to 1400 G/L) to the 50 AML patients with the lowest maximum platelet values (range 10 to 120 G/L) after the first induction cycle (Table 3). We assessed thrombopoietin protein (TPO) levels in plasma and protein levels of the thrombopoietin receptor MPL in mononuclear cell extracts (Fig. 3A) both obtained at diagnosis before initiation of treatment. A higher plasma TPO level at diagnosis was the most significant marker to indicate EPR (p < 0.0001), while MPL protein levels in the mononuclear cell extracts did not differ between the two groups. We also analyzed mRNA expression of the thrombopoietin receptor gene *MPL* in patient samples collected at diagnosis, as well as mRNA expression of transcription factors involved in thrombopoiesis including the megakaryocyte inhibitor *MYB* and the thrombopoietic factors *GATA1* and *NFE2*. Myeloid transcription factors crucial for monocyte differentiation including *GATA2*, *KLF4,* and *NF1A* were similarly investigated.

**Table 3:**
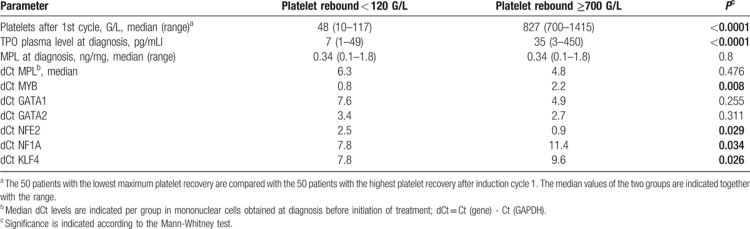
Expression of genes involved in megakaryopoiesis in the patients with minimum vs maximum platelet recovery after the first induction cycle

**Figure 4:**
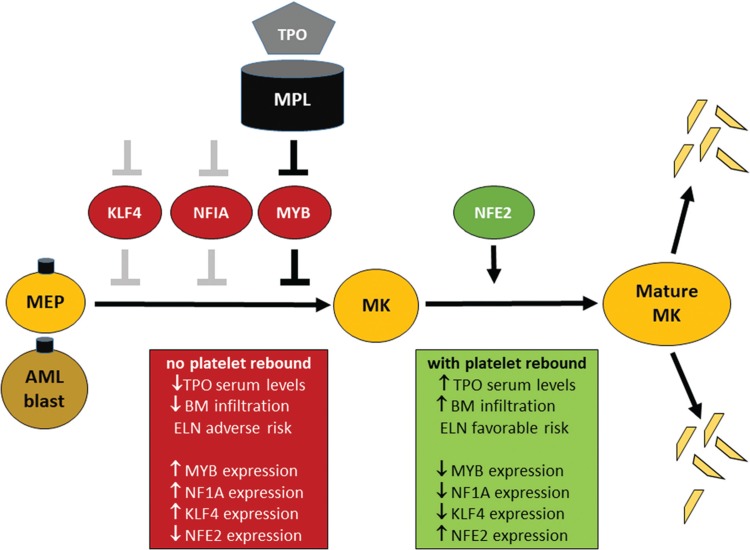
Excess platelet rebound above 500 G/L after induction cycle 1 (EPR) after high dose chemotherapy correlates to inverse mRNA expression levels of the hematopoietic transcription factors *KLF4*, *NFIA*, and *MYB* and is associated with *NFE2* mRNA expression in mononuclear cells at diagnosis of AML. Inhibitory functions of transcription factors in the megakaryocyte-erythrocyte precursor cell (MEP) are hypothetical for *KLF4* and *NFIA* (grey bars) and evidence-based for *MYB* (black bars). Activating functions are evidence-based for *NFE2* (black arrow), a differentiation inducer in megakaryocytes (MK).

We observed significant differences in *NFE2* gene expression between both groups. In contrast, we found no correlation of EPR with expression levels of *MPL, GATA1,* and *GATA2* in mononuclear cells at diagnosis. There were negative correlations of EPR with expression levels of *MYB*, *KLF4,* and *NF1A* genes, with significant differences of expression levels of all 3 markers between both groups (Fig. 3B and Table 2). These observations are summarized in a model as depicted in Figure 4.

## Discussion

In this study, we investigated the dynamics of platelet recovery after the first standard chemotherapy induction cycle in 291 newly diagnosed AML patients treated at a single academic center. We found that excess platelet rebound (EPR+) defined as exceeding 500 G/L is a common event identified in 120 patients (41.2%). EPR+ was predominantly observed in ELN favorable risk AML, and it was associated with significantly better overall and disease-free survival as compared to patients with platelet levels below the respective threshold. In addition, EPR+ represented an independent prognostic parameter for improved survival by multivariate analysis. Interestingly, a threshold of platelets of 500 G/L or more was best suited to define patients with more favorable outcome.

Remarkably, the observation of EPR after the first induction cycle appears to be a hallmark of favorable risk AML patients. In particular, ELN favorable risk subgroups consistently had more often EPR after cycle 1 compared to intermediate-I or adverse risk subgroups. In particular, patients with t(15;17)/*PML-RARA*, inv(16)/*CBFB-MYH11*, t(8;21)/*RUNX1-RUNX1T1* or *NPM1* mutations (with *FLT3* wild type) achieved EPR in more than 50% of cases, whereas EPR was a very rare event in patients with chromosome 7 alterations or complex abnormalities. Although the reasons remain unclear, one may speculate that the more rapid blast clearance in patients with favorable risk already after the first induction cycle allows a more pronounced platelet recovery. Strikingly, EPR never occurred in patients with therapy-related AML.

Aiming to unravel the underlying pathophysiologic mechanisms, we performed measurements of some growth factors, receptors, and transcription factors with known involvement in megakaryo- and thrombopoiesis in the plasma and in mononuclear cells obtained at diagnosis before chemotherapy. First, a higher plasma thrombopoietin (*TPO*) level at diagnosis was a reliable marker to indicate the occurrence of EPR (p < 0.0001) after the first induction cycle, whereas we observed no differences in MPL protein levels in mononuclear cells. In addition, we found a positive correlation of EPR with higher mRNA expression levels of *MPL*, *GATA1*, *GATA2,* and *NFE2* genes in mononuclear cells at diagnosis, whereas expression levels of *MYB*, *KLF4,* and *NF1A* genes were inversely correlated. Thrombopoietin (TPO) is considered the master regulator of megakaryopoiesis. TPO is predominantly exerting its downstream effects during megakaryopoiesis as well as its upstream effects on hematopoietic stem cells through its receptor MPL.^24–29^ TPO levels are largely regulated by MPL receptor-mediated cytokine-scavenging, with platelets and megakaryocytes acting as the primary scavengers.^30–31^ Previous work has suggested that AML cells can express MPL,^32^ and that TPO can promote growth of leukemic cells in vivo.^33^ Leukemic cells expressing high levels of MPL were shown to clear TPO, thereby leading to insufficient cytokine levels for non-leukemic hematopoiesis.^20^ Consequently, MPL expression was identified to act as a central predictor of thrombocytopenia in AML patients at diagnosis.^20^ Interestingly, these studies suggested a lack of correlation between bone marrow blast infiltration and cytopenias in AML at diagnosis.

Noteworthy, we observed that MPL protein expression in mononuclear cells at diagnosis did not differ between patients with or without EPR after induction cycle 1. In contrast, TPO plasma levels at diagnosis were significantly higher in EPR+ patients after first induction compared to patients without EPR. These data suggest that differing dynamics between TPO and MPL are involved at diagnosis and during hematologic recovery after induction chemotherapy. Whereas high MPL expression in leukemic cells at diagnosis of AML can lead to MPL receptor-mediated cytokine-scavenging of TPO and to pronounced thrombocytopenia independent of the degree of bone marrow infiltration, the occurrence of EPR after induction treatment seems to be independent of MPL expression on leukemic cells at diagnosis, but strongly associated with TPO plasma levels at diagnosis. In addition, AML patients with EPR in our cohort had higher degrees of bone marrow infiltration at diagnosis and lower platelet counts at diagnosis. Thus, high TPO levels at diagnosis in EPR+ patients (probably due to increased bone marrow infiltration) appear to lead to increased stimulation of megakaryopoiesis thereby translating into EPR during hematologic recovery after blast clearance in induction cycle 1.

In summary, our study demonstrates that the achievement of excess platelet rebound of 500 G/L or more following first induction chemotherapy is strongly associated with more favorable survival outcomes and with some specific favorable genetic subgroups of AML. In contrast, patients with ELN intermediate or adverse risk typically failed to show EPR. Interestingly, we could demonstrate that the achievement of EPR after the first induction chemotherapy was correlated with higher plasma thrombopoietin (TPO) level at diagnosis, as well as with upregulation of some specific transcription factors involved in megakaryocyte differentiation such as *NFE2*. Future research may aim at further clarifying the background of hematologic recovery as this may facilitate the clinical interpretation of the early course of patients with AML during induction chemotherapy.

## Acknowledgments

The authors wish to thank all staff members involved in the care of the patients reported in this study.

## Supplementary Material

Supplemental Digital Content
