# Supplementary material for: Rebound Thrombocytosis after Induction Chemotherapy is a Strong Biomarker for Favorable Outcome in AML Patients
Source: Hemasphere. 2019 Mar 20;3(2):e180. doi: 10.1097/HS9.0000000000000180 (PMC6746035; doi:10.1097/HS9.0000000000000180)
Supplement: Supplemental Digital Content [file hs9-3-e180-s001.pdf]

Supplementary Figure 1

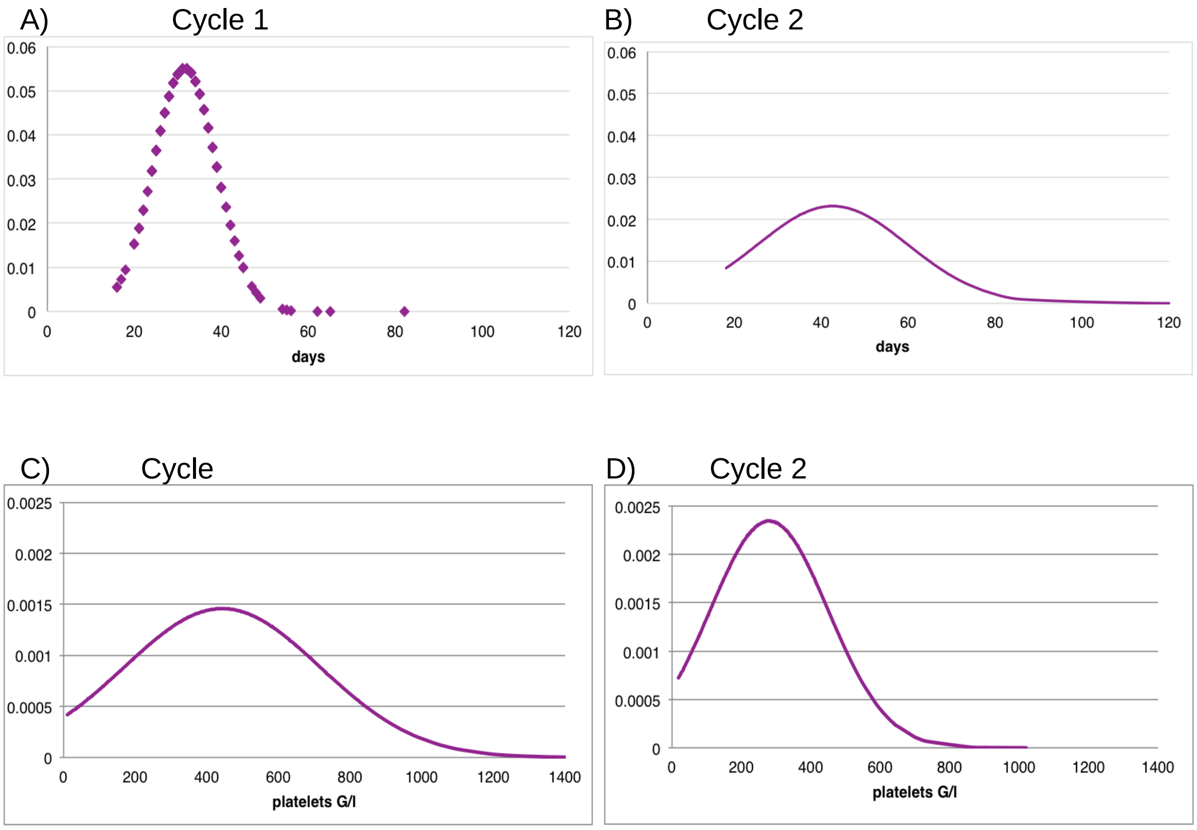

The two top panels indicate when maximum platelet recovery was observed in the AML patients in this study after induction cycle 1 (**A**) and cycle 2 (**B**). X-axis are days since the first day of chemotherapy of the cycle, and Y-axis is percentage of all patients (e.g. 0.01 = 10%). The lower panels indicate the range of maximum platelet recovery of all patients in this study after induction cycle 1 (**C**) and cycle 2 (**D**). X-axis depict maximum platelet values, and Y-axis is percentage of all patients.

Supplementary Figure 2

Favorable ELN risk group

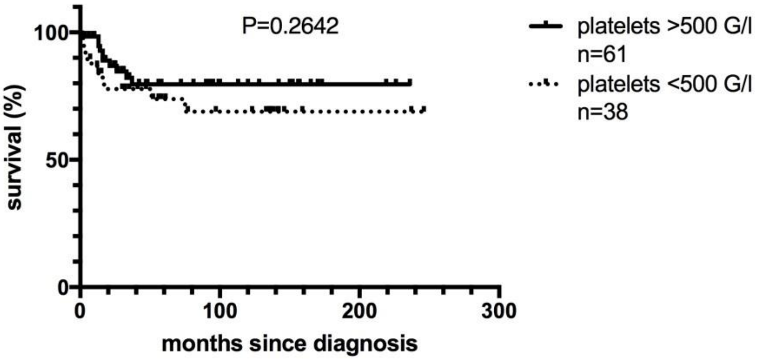

intermediate-II risk group

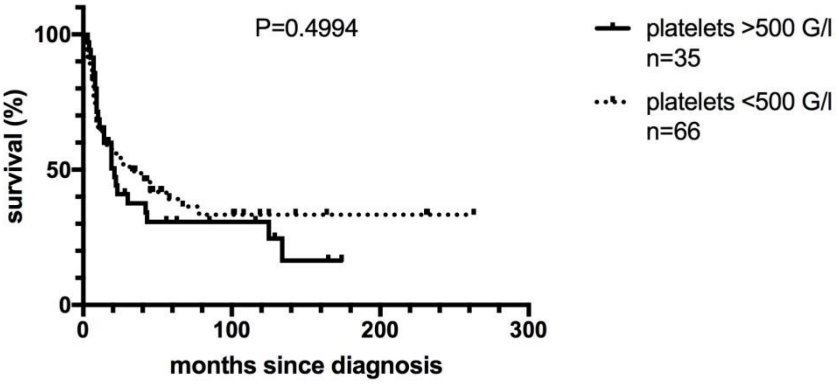

intermediate-I risk group

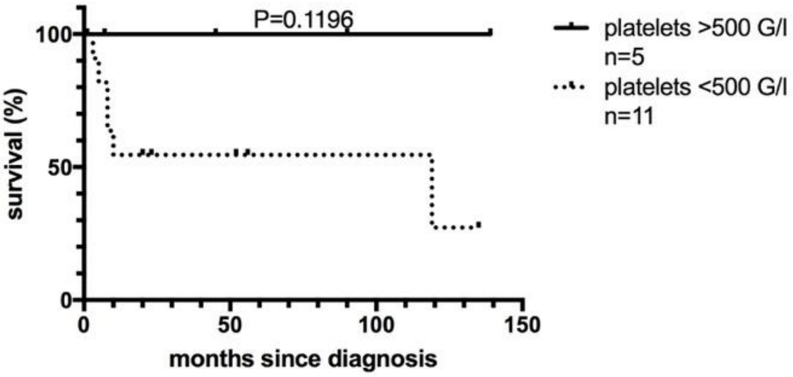

Adverse risk group

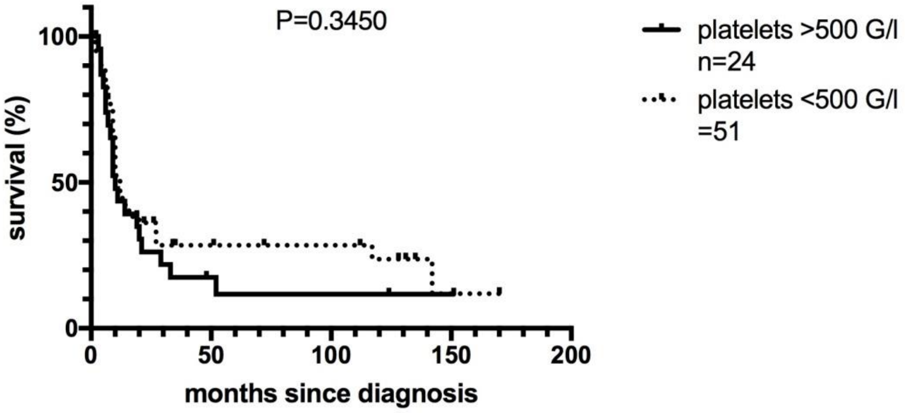

Overall survival is shown for the four ELN (European Leukemia Net) risk groups comparing AML patients with versus without excess platelet rebound (EPR) after the first induction cycle within each risk group.

Supplementary Figure 3

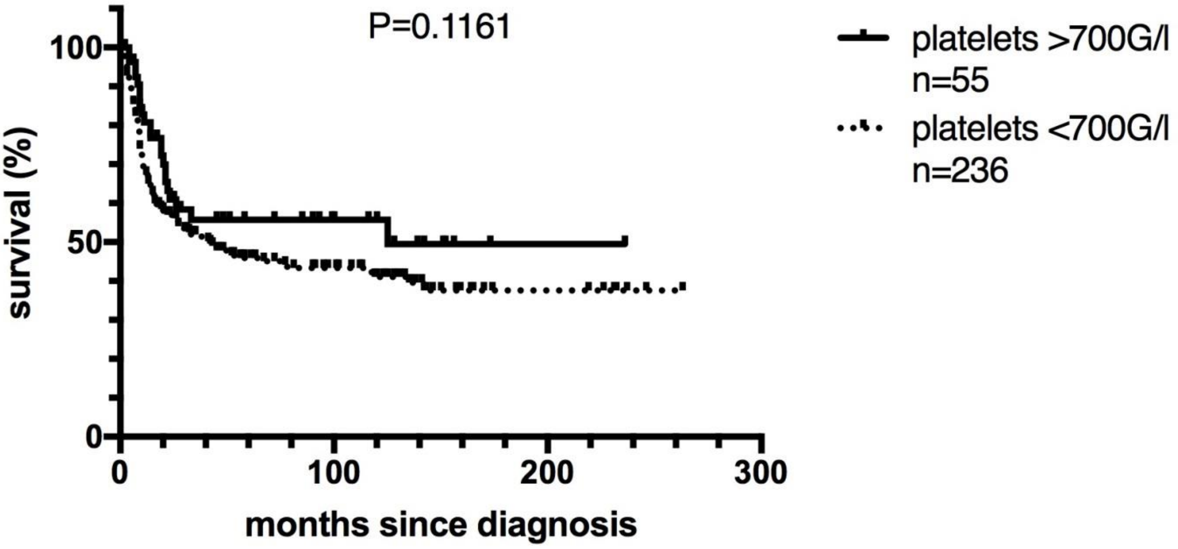

Overall survival is presented comparing AML patients with versus without platelet rebound using a platelet threshold of 700 G/L after the first induction cycle.
